# Multiplex engineering and multifunction T cells for precise and effective immunotherapies

**DOI:** 10.3389/fimmu.2025.1680410

**Published:** 2025-10-16

**Authors:** Leila Jafarzadeh, Ali Smaani, Jean-Sébastien Delisle

**Affiliations:** ^1^ Centre de Recherche de l’Hôpital Maisonneuve-Rosemont (CRHMR), Montréal, QC, Canada; ^2^ Institut d’hématologie-Oncologie et Thérapie Cellulaire, Montréal, QC, Canada; ^3^ Department of Medicine, Université de Montréal, Montréal, QC, Canada

**Keywords:** adoptive immunotherapy, genetic engineering, cancer immunotherapy, T cells, CRISPR-Cas9, chimeric antigen receptor, Transgenic TCR

## Abstract

Adoptive T cell transfer has emerged as a pillar of modern cancer immunotherapy. Propelled by viral and non-viral-based technologies, such as CRISPR-Cas9, genetic engineering offers novel opportunities for both emerging cellular therapies and the improvement of more established approaches such as chimeric antigen receptor (CAR) modified T cells. First-generation genetically modified T-cell therapeutics remain limited by the intrinsic constraints imposed by T-cell biology, such as T-cell exhaustion, poor trafficking into hostile tumor beds, toxicity, and challenges associated with tumor antigenic escape. Several of such limitations can be addressed by further engineering, expanding significantly the potential of cell therapy. This review focuses on the promise of using currently available cellular engineering technologies to genetically engineer single T cells at multiple different loci and/or confer several novel functions to circumvent the shortcomings of adoptive immunotherapy to treat cancer. Various methodologies and rationales for the design of these advanced engineered cellular products are described, along with emerging clinical data supporting the use of multiplex-engineered T cells. The limitations of advanced cell engineering and the remaining gaps that need to be filled to optimize the efficacy of adoptive T-cell immunotherapies are also discussed.

## 1 Introduction

T-cell transfer to treat cancer was pioneered through allogeneic hematopoietic transplantation (AHCT) in the 1970s, followed by tumor-infiltrating lymphocyte (TIL) therapy and other *ex vivo* expanded antigen or pathogen-specific T-cell products (1–3). Although therapies using unmodified T cells remain highly relevant today, the development of multiple gene engineering tools to impart novel or improved functions to immune cells is transforming the field. The rapid and widespread adoption of autologous chimeric antigen receptor (CAR)-modified T cells for the treatment of B-cell malignancies reflects this transformative potential. Through synthetic biology, CAR T cells can recognize cell surface proteins outside the major histocompatibility complex (MHC) context, adding a new dimension to the use of T cells as therapeutic agents. In parallel, the ever-expanding definition of the MHC ligandome in several cancers has enabled the discovery of cancer-specific T-cell receptors (TCR) that can be used for transgenic TCR T-cell therapies (4–7). Despite this remarkable progress, both unmodified T cells and currently approved CAR or transgenic TCR T cells face limitations. For example, autologous CAR T cells approved for the treatment of B-cell malignancies are toxic and can fail because of either intrinsic T-cell dysfunction and poor persistence or because neoplastic cells evolve to suppress the expression of the target antigen. Furthermore, they are costly and require complex logistics to deliver treatment on time. The use of allogeneic T cells can, in principle, address several shortcomings of autologous therapies by manufacturing large batches of ready-to-use “off-the-shelf” products from healthy donors. However, it is limited by bidirectional alloreactivity that can lead to adoptively transferred T-cell rejection and nonspecific host tissue damage in the form of graft-versus-host disease (8). Several of these shortcomings can be addressed through multiplex gene engineering, whereby several modifications are incorporated into individual T cells to improve their functionality in different contexts. This can be achieved through various approaches, including polycistronic vectors that enable simultaneous expression of multiple transgenes (single engineering step to impart multiple functions) or more modular strategies that combine different individual gene edits (multiplex engineering) to consolidate a single function (e.g., targeting multiple antigens) or to confer different functions (e.g., specific antigen targeting and resistance to T-cell exhaustion). Herein, we review various approaches that can be used to perform multigene editing and/or confer multiple novel functions to therapeutic T- cells and describe how they can be applied to T-cell therapy. Three main themes will be developed: 1) multi-engineering to address the limitations of T-cell biology, 2) multi-engineering to better engage cancer cells, and 3) multi-engineering to prevent excessive toxicity. Finally, published clinical study reports in which multiplex gene-engineered therapeutic products were used will be reviewed.

## 2 Methodologies for T-cell engineering

**Figure 1:**
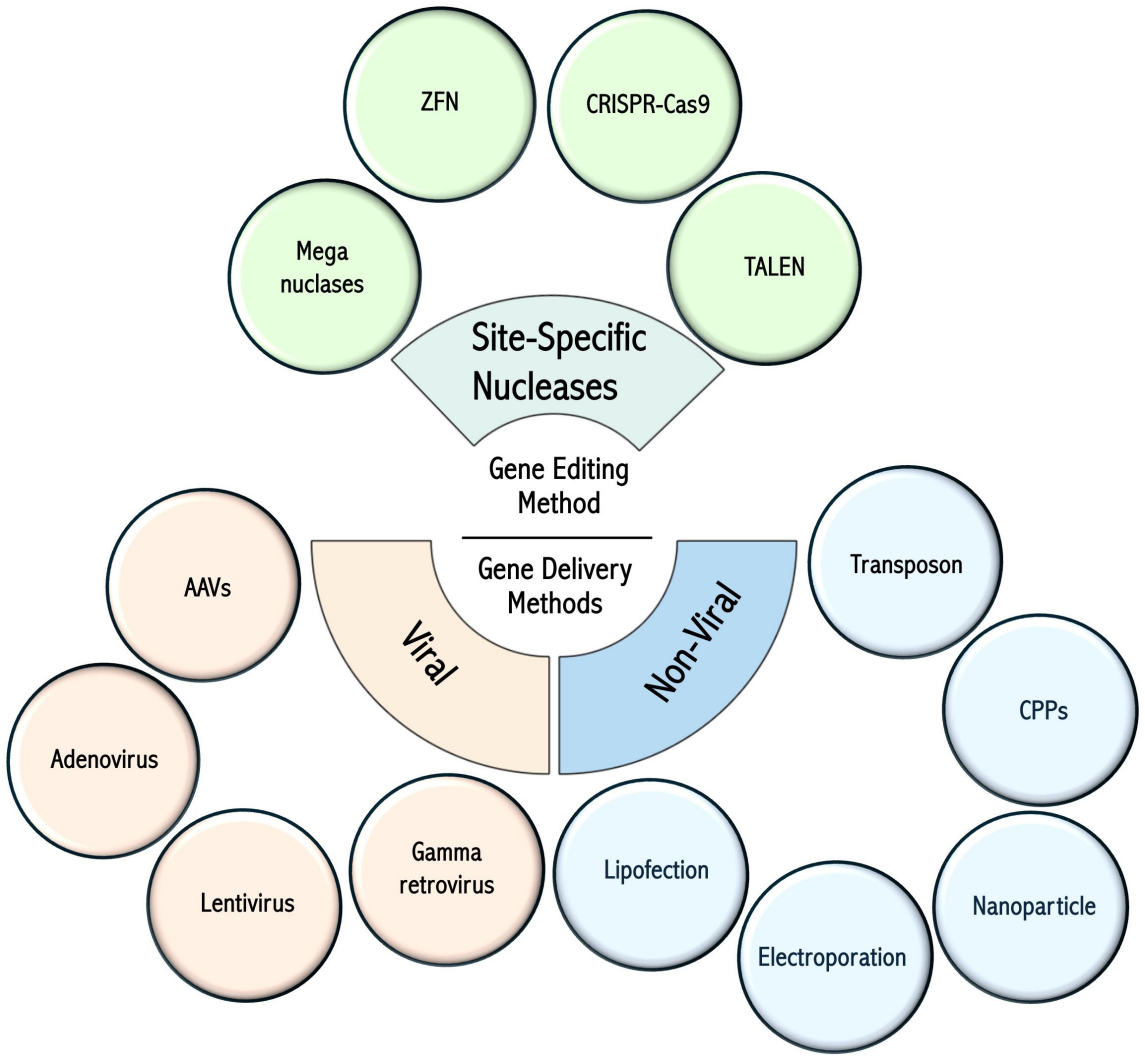
Gene editing technologies and cell delivery methods. Summary of the various gene engineering methods used for T-cell modification. Figure constructed with visual elements from BioRender (https://BioRender.com/qtcywde).

Various methods for the genetic modification and engineering of T cells include approaches to insert exogenous genetic material (e.g., transgenes) into the genome or alter the sequence of a given gene to modulate expression or modify protein sequence. Other strategies involve the transfer of non-genome-integrating genetic material (plasmids, mRNA) to confer novel functions to T cells (9–11). T cell gene engineering methods can be broadly assigned to two main categories: those that involve the non-targeted insertion of genetic material into the host genome (viral vectors, transposons, etc.) and targeted gene editing technologies, which involve site-specific nucleases such as meganucleases (12), Zinc Finger Nucleases (ZFN) (13), Transcription Activator-Like Effector Nucleases (14) (TALEN), and clustered regulatory interspaced short palindromic repeats (CRISPR)-Cas9-based technologies (15). Non-targeted methods leverage natural viral and non-viral mechanisms, permitting the integration of genetic material into host genomes at multiple loci. These include (gammaretroviruses (16) and lentiviruses (17)), and transposon-based systems such as Sleeping Beauty (18) and PiggyBac (19). Other viruses, such as adenoviruses (20) and adeno-associated viruses (AAV) (21) can deliver genetic material into cells, and non-viral methods are increasingly used to deliver various cargoes (nucleic acids, proteins, etc.). The delivery of these cargoes relies on various methods, such as lipofection (22), electroporation (23), nanoparticles (24), and cell-penetrating peptides (CPPs) (25, 26). The methodologies for gene editing and intracellular delivery, as well as their respective advantages and disadvantages, are summarized in **Figure 1** and **Table 1**.

**Table 1:** Advantages and disadvantages of different gene engineering approaches.

Methods	Advantages	Disadvantages
Site-Specific Nucleases (Targeted)	- Permanent edits (CRISPR-Cas9, ZFN, TALEN) - High efficiency (CRISPR-Cas9, Base/Prime editors) - No DSBs (Base/Prime editors)	- Genomic instability/off-targets (CRISPR-Cas9, ZFN, TALEN) - Imprecise repair via NHEJ (CRISPR-Cas9, ZFN, TALEN) - Chromosomal translocations (CRISPR-Cas9, ZFN, TALEN) - Large size delivery issue (TALEN, Prime editors) - Complex design (ZFN, TALEN, Meganuclease)
Viral Delivery	- Stable expression (Gammaretrovirus, Lentivirus) - Infect non-dividing (Lentivirus, Adenovirus) - High efficiency (Gammaretrovirus, Lentivirus, Adenovirus, AAV) - Safer integration (Lentivirus) - Transient expression (Adenovirus, AAV)	- Insertional mutagenesis (Gammaretrovirus, Lentivirus) - Require dividing cells (Gammaretrovirus) - Immunogenicity (Adenovirus) - Limited cargo (AAV) - Cost (Gammaretrovirus, Lentivirus, Adenovirus, AAV)
Non-viral Delivery	- Low immunogenicity (Electroporation, Lipofection, Nanoparticle, CPPs) - Simple, low-cost (Electroporation, Lipofection, Nanoparticle, CPPs) - Large cargo capacity (Electroporation, Nanoparticles, CCPs, Transposons) - Transient expression (Electroporation, CCPs) -Stable expression possible (Transposons)	- Lower efficiency (Lipofection, Nanoparticle, CPPs) - Cytotoxicity (Electroporation, Lipofection) - Equipment needed (Electroporation) - Limited *in vivo* applicability (Electroporation, Lipofection, Nanoparticle, CPPs)

Clustered regularly interspaced short palindromic repeats (CRISPR), Zinc-finger nuclease (ZFN), Transcription activator-like effector nuclease (TALEN), Double-stranded breaks (DSB), Non-homologous end-joining (NHEJ), Adeno associated virus (AAV), Cell penetrating peptide (CPP).

### 2.1 Genetic modification tools

Meganucleases, or homing endonucleases, are early gene-editing tools that recognize long DNA sequences (14–40 bp) with high specificity and minimal off-target effects (12); however, they are difficult to reprogram for new target sequences (27). Engineered variants, such as megaTALs (TALE fused to meganucleases), have been applied to T-cell editing, such as T-cell receptor alpha constant (TRAC) region disruption. Zinc-finger nucleases (ZFNs), introduced in 1996 (28), combine zinc finger DNA-binding domains with FokI nucleases to induce targeted DNA breaks (29). They showed genome-editing potential in eukaryotic cells (30) and were used for CCR5 disruption in human T cells (31). However, ZFNs are complex and costly to design (32–34), leading to the development of TALENs in 2010 (14, 35). TALENs recognize individual nucleotides with high precision, target longer sequences than ZFNs, and offer a simpler design; however, their large size (~3 kb) limits their multiplex gene-editing applications (36).

Clustered Regularly Interspaced Short Palindromic Repeats (CRISPR)-Cas9 has emerged as a flexible and scalable gene-editing system that enables efficient multiplex editing via a simple guide-RNA (sgRNA) design. Discovered as bacterial repetitive sequences (37) and later identified as part of bacterial adaptive immunity (38), CRISPR-Cas9 enables the RNA-guided targeting of almost any DNA sequence with high efficiency (15). Unlike ZFNs and TALENs, CRISPR-Cas9 does not require target-specific protein engineering and induces double-strand breaks (DSBs) that are repaired by cellular pathways such as non-homologous end joining (NHEJ) or homology-directed repair (HDR) (39). Thus, it remains a promising tool for precise multiplex genome engineering (39, 40). However, CRISPR-Cas9-induced DSBs can cause off-target mutations and chromosomal translocations, compromising genome integrity (41) (see quality control considerations below). NHEJ is error-prone and reduces precision (42), whereas HDR is inefficient, particularly in non-dividing cells. Strategies to enhance HDR include tumor suppressor p53-binding protein 1(53BP1) inhibition and RAD18 (Radiation-sensitive 18) (43, 44), modified CRISPR systems, small molecules modulating DNA repair, and co-localization of repair templates with Cas9 (45).

In 2016, base editing was introduced by Liu et al. to enable precise single-nucleotide changes without DSBs or donor DNA templates. Cytosine and adenine base editors allow direct base conversions and reduce genomic rearrangements (46), although they are limited to transitions and are prone to off-target deamination and bystander editing. Prime editing (PE) was developed to address these limitations, enabling diverse substitutions, small insertions, and deletions without DSBs using Cas9 nickase fused to reverse transcriptase and pegRNA to direct edits (47). Despite their high precision, challenges include low efficiency in some cells, large construct sizes, and pegRNA mispriming (47). Prime editing evolved from PE1 with natural reverse transcriptase to PE2 using engineered enzymes, and PE3 added a second nick to increase efficiency but with increased indels, which PE3b mitigated by timing the second nick (48). PE4 and PE5 further enhance precision with DNA repair inhibitors (49), and enhanced Prime Editors (ePE) improve pegRNA stability and editing yield (50). These developments have progressively improved the efficiency, specificity, safety, and suitability of prime editing for multiplex gene-editing applications.

In addition to DNA-targeting tools, RNA-targeting technologies, such as CRISPR-Cas13, have emerged as powerful alternatives for modulating T-cell function without permanent genomic changes (51). Cas13 cleaves single-stranded RNA transcripts to transiently and reversibly regulate gene expression (52). In a striking demonstration of Cas13-based knockdown possibilities in T cells, Tieu et al. revealed that the co-transduction of Cas13d and multiple sgRNAs could reduce the expression of multiple target genes simultaneously, enabling the suppression of multiple immune checkpoints or entire metabolic pathways (53). This system can also be used for combinatorial screens and can be modified to permit drug-controlled Cas13d expression and graded target gene suppression, which may be advantageous over complete ablation in certain settings. Catalytically inactive Cas9 (dCas9) can be fused to transcriptional activators, leading to specific gene expression (CRISPR activation) in CAR T cells (54) without altering the DNA sequence at the targeted loci. The fusion of dCas9 E to epigenetic modulators enables targeted chromatin remodeling and gene expression changes without DNA breaks (55, 56). Early studies in primary T cells revealed that this approach could stabilize Foxp3 expression in mouse regulatory T cells and delay replicative senescence in stimulated human T cells through the expression of telomerase reverse transcriptase (TERT) (57, 58). Although all are at the pre-clinical stage, these RNA- and epigenetic-targeting approaches offer great promise for the precise control of T cell phenotypes while reducing the risks associated with permanent genomic alterations.

### 2.2 Gene editing delivery methods

The insertion of new genetic material or genome editing requires the delivery of different cargoes, depending on the method used. Gene delivery methods can be classified into viral and non-viral approaches, each with specific advantages and disadvantages. Gammaretroviruses allow stable integration but require dividing cells to do so. While insertional mutagenesis is a theoretical concern for any retroviral vectors, experimental evidence in mature T lymphocytes suggests that these cells are relatively resistant to transformation (59). Long-term follow-up studies of patients treated with gammaretroviral-modified T cells have not reported malignant transformation (60, 61). Lentiviruses, a retroviral subclass, can transduce both dividing and non-dividing cells (62) and support stable gene expression, in addition to enabling multiplex gene editing (63) and the insertion of large polycistronic constructs. Lentiviral vectors offer key advantages for CAR T-cell therapy, including efficient T-cell transduction, durable expression, and a safer integration profile than gammaretroviruses (64). Third-generation lentiviral systems further enhance safety by separating viral components and using self-inactivating elements (65, 66). Importantly, no significant genotoxicity or malignant transformation has been reported in clinical CAR T-cell applications using lentiviral vectors (67), although concerns regarding insertional mutagenesis remain (68).

Adenoviruses are non-integrating viruses in the host genome and provide transient gene expression. However, they can elicit strong immune responses directed against the viral vector, which may limit their therapeutic efficacy (69). AAVs are less immunogenic and can support longer transgene expression durations; however, they are limited by their small cargo capacity and high production costs (70). Although less of a concern for other cellular engineering approaches, the risk of immunogenicity is a preoccupation whenever a foreign (natural or synthetic) molecule is introduced into a therapeutic cellular product. Cas9 nucleases (bacterial proteins) can elicit T-cell and humoral responses, and pre-existing immunity is prevalent in the population (71, 72). Despite the possibility of reducing immunogenicity through protein engineering (73), current clinical protocols using Cas9 modified T cells insist on transient exposure to Cas9 and the absence of the protein in the final product. Synthetic proteins (artificial receptors for example) may also be recognized as non-self and impact the persistence of the transferred T cells (74, 75). Even if heavily treated cancer patients may not be able to mount immune responses against foreign proteins as well as normal individuals, multiplex editing and/or the introduction of multiple artificial transgenes could increase the risk of early rejection.

Transposon-based systems, such as Sleeping Beauty, offer non-viral, nuclease-free integration with low cost and large cargo capacity (18, 76–78). However, they face limitations in transfection efficiency, delivery synchronization, and the risk of semi-random integration (79, 80).

Non-viral methods such as electroporation, nucleofection, CPPs, lipofection, nanoparticles, and transposons offer low immunogenicity, simplified production, and reduced biosafety risks (10, 23, 81–83), but often result in lower efficiency and transient expression, unless paired with integration systems. Among non-viral methods, electroporation and nucleofection are efficient for delivering genetic material into primary T cells, supporting the simultaneous delivery of multiple components (84, 85). This makes them ideal for multiplex editing strategies in T-cell engineering. Electroporation is a widely used method for delivering ribonucleoproteins (RNPs) or mRNA for CRISPR-based gene editing, allowing the effective delivery of Cas proteins and gRNAs without viral vectors (86). Despite being scalable to suit clinical purposes and yielding a high number of genetically modified T cells, electroporation and nucleofection can lead to significant cytotoxicity, especially when applied to minimally cultured or naïve T cells, potentially compromising the quality of the final product (10, 23, 83). Editing is typically performed prior to, or early after, T-cell activation (within 24–48 h) to maximize repair efficiency and viability (87, 88). This is important when both CRISPR-Cas9 and gammaretro-lentiviral methods are used on the same T cells for multiplex engineering purposes. Early editing with CRISPR-Cas9 avoids the cleavage of integrated vectors if CRISPR targets overlap with viral vector sequences (88). Recent clinical studies have highlighted that editing resting or minimally activated T-cells reduces chromosomal abnormalities linked to DSBs, supporting carefully timed editing workflows for safety and efficacy in clinical manufacturing (see Section 2.3). Another non-viral method for gene delivery into T cells is the use of CPPs which are short peptides that can traverse cell membranes and facilitate the intracellular delivery of various cargos, including nucleic acids and proteins. This approach has been investigated for the delivery of CRISPR/Cas9 components into T cells. For example, CPPs such as PepFect14, LAH5, TAT peptide, Transportan-10, and MPG have been successfully applied to deliver CRISPR/Cas9 plasmids or RNP complexes into primary human T cells (89–92). Although promising, CPP-mediated delivery still faces challenges, such as potential cytotoxicity, limited efficiency compared to viral or electroporation-based methods, and the need for optimization to achieve robust genome editing in clinical-grade T cell products (93–95). Hence, combining gene-editing tools (e.g., CRISPR-Cas9, base editors, transposons) with gene delivery platforms, such as lentiviral vectors, allows the creation of customized multi-edited or multi-functional T-cell products.

### 2.3 Genotoxicity and quality control in T-cell gene engineering

Multiplex gene editing in T cells offers substantial therapeutic potential but also raises significant concerns about genotoxicity due to the induction of multiple DNA double-strand breaks (DSBs). These breaks can lead to chromosomal translocations, large deletions, and complex rearrangements, such as chromothripsis, which compromise genome stability, reduce cell viability, and may even induce transformation or cancer (88, 96). Several strategies have been developed to reduce the risk of genotoxicity. These include the use of nucleases with different cutting patterns (such as Cas9 and Cas12a/b), temporal separation of sgRNA delivery, and favoring ribonucleoprotein (RNP) delivery over plasmids to limit the active time of nucleases. Promoting HDR over NHEJ, carefully designing sgRNAs, and transiently inhibiting NHEJ using small molecules, such as NU7441, can further improve genomic safety (96). Additionally, studies have shown that performing gene editing within 24 to 48h after T cell activation reduces p53-dependent DNA damage responses, thereby enhancing editing efficiency and cell recovery (88). It is also important to consider the risk of damaging the integrated vector sequences when combining multiplex editing and lentiviral transduction. Cas9 activity near or within a vector can disrupt transgene expression or cause loss of function (88). Therefore, delaying transduction until 48–72 hours post-editing is often beneficial, although this must be balanced by the activation status and susceptibility of T cells to infection. To evaluate the genotoxic impact of the editing process, assessment of cellular stress and DNA damage response (DDR) markers, such as p53, γH2AX, and apoptosis indicators, is essential, as DDR plays a central role in detecting and repairing DNA damage, maintaining genome stability, and preventing mutagenesis and tumorigenesis (97). Chromosomal rearrangements caused by CRISPR-Cas9-induced double-strand breaks (DSBs), especially during multiplex editing with multiple sgRNAs, are concerning (96). To mitigate these risks, high-fidelity Cas9 variants and novel editing platforms, such as base and prime editors, are being actively explored. Base editing enables precise nucleotide substitutions without double-strand breaks (DSBs), reduces genotoxic risks such as deletions, translocations, and p53 activation (46, 98), and is especially suited for multiplex or subtle edits. Off-target effects are also a major consideration in both CRISPR and base editing technologies, and require comprehensive assessment using techniques, such as GUIDE-seq, CIRCLE-seq, or deep whole-genome sequencing to evaluate specificity (99, 100).

This review focuses on multiplex gene editing, as it is increasingly moving into the clinical stage. Hence, safety concerns must be stratified and addressed according to the number and type of edits. CRISPR-Cas9-mediated double-strand breaks (DSBs) can activate the p53 pathway, induce chromosomal translocations, and drive immune responses, with the frequency of deleterious events increasing when multiple loci are targeted (101). Base and prime editors reduce DSB-related risks but will require long-term surveillance to monitor low-frequency off-target effects (102). Long-term surveillance is required to monitor the potential clonal expansion of edited T cells. The maximum number of gene edits or off-target lesions that a T-cell can sustain before functional impairment or death is unknown and likely depends on the genes being targeted. However, “over-engineering” remains a theoretical issue. Strategies that do not induce double-stranded DNA breaks, such as base and prime editing, appear to be ideal for the genetic editing of multiple genes through a single engineering step. Multiple gene knockdown with limited genetic risk may also be achieved using traditional viral vectors encoding multiple miRNAs (103) and Cas13-based approaches (as described above).

However, viral vectors require vigilance and well-designed quality control (QC) strategies. One key parameter is the vector copy number (VCN), as high VCNs (>5–10 copies per genome) in lentiviral and gammaretroviral systems are linked to an increased risk of insertional mutagenesis and oncogene activation (104). Therefore, clinical protocols aim for a VCN of 1–5 copies per cell to balance transgene expression with genomic safety (105). Additionally, verifying full-length transgene integration and expression is essential when using viral vectors, which is typically assessed using digital droplet PCR (ddPCR), long-read sequencing, or functional assays (106). These assays are usually required to complete other QC assessments necessary to evaluate the identity, purity, potency, and sterility of the final cellular product. The regulatory landscape for cell and gene therapies is rapidly evolving and can vary according to jurisdiction, mandating careful planning of quality control strategies by advanced T-cell product developers.

## 3 Multiplex-engineering to address the limitations of adoptive T-cell immunotherapy

**Figure 2:**
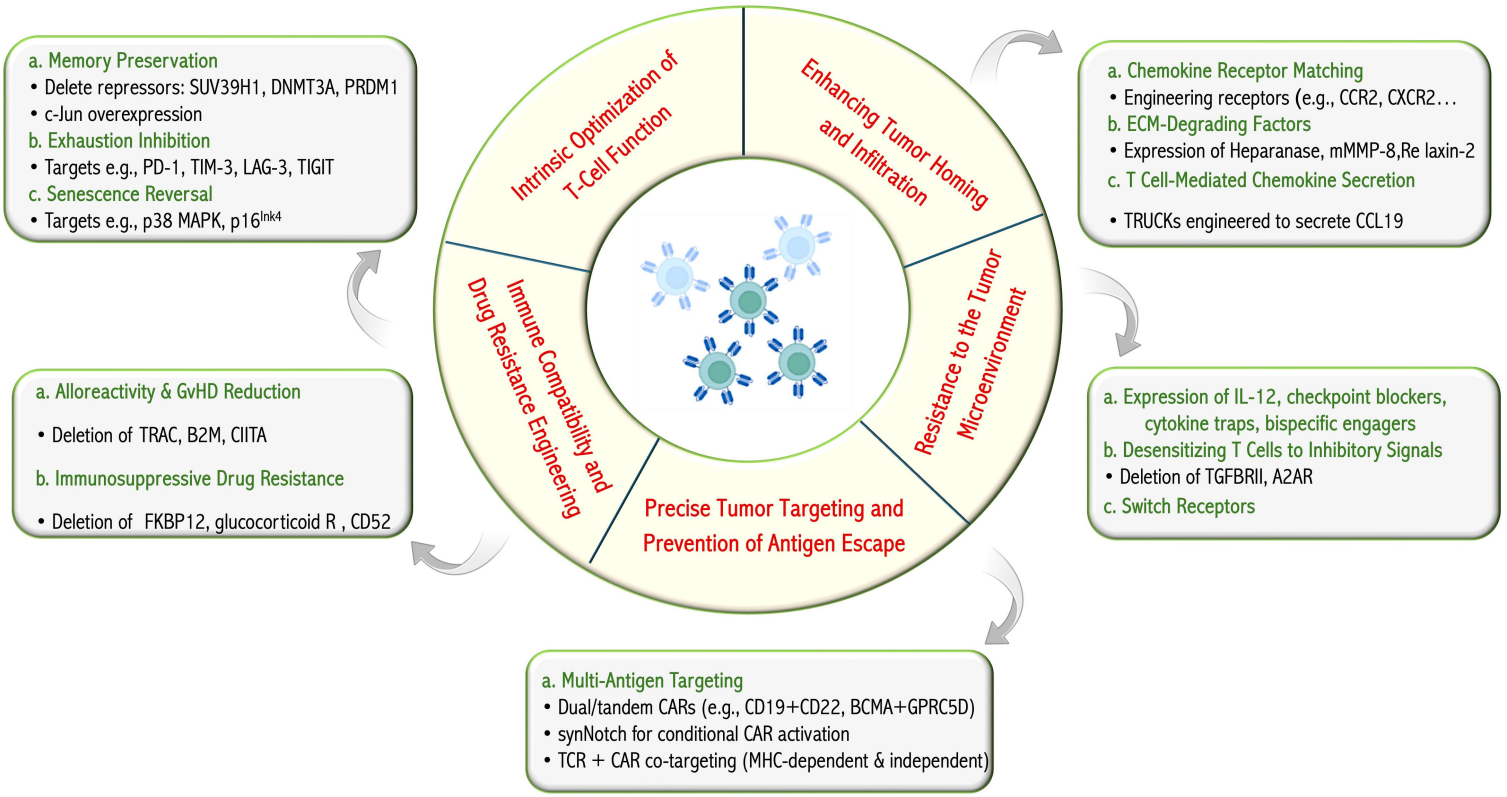
T-cell engineering for improved cancer targeting. The therapeutic and biological objectives pursued through T-cell engineering and a summary, with examples, of strategies integrated in multifunction T cells reported in pre-clinical or clinical studies. Figure constructed with visual elements from BioRender (https://BioRender.com/h5481mb).

Effective adoptive T-cell immunotherapy depends on the intrinsic quality of T-cells, which is influenced by several factors, including the previous treatments received by the patient (autologous therapies), the manufacturing process, and the context following adoptive transfer (repeated antigen exposure leading to exhaustion, homeostatic cytokine availability, and tumor microenvironments) (107, 108). Here, we discuss the requirements for effective and safe cancer T-cell therapy, including T-cell fitness, effective recognition of cancer cells, function within cancer microenvironments, and mitigation of immune-related adverse effects. We specifically reviewed the strategies involving multi-engineering to address one or several of these requirements for effective tumor eradication (**Figure 2**).

### 3.1 Addressing the limitations of T-cell biology

#### 3.1.1 T-cell dysfunction

Conventional T cells develop as long-lived cells, each bearing a unique TCR, and are responsible for the detection of infected and transformed cells through TCR-mediated recognition of MHC-associated peptides. The activation and further differentiation of T cells are influenced by several other signals, including co-stimulation, cytokines, and metabolites (108). Upon repeated antigenic exposure, depending on the context, T cells develop features of terminal effector differentiation and loss of memory potential, senescence, and/or exhaustion, which limit their efficacy. A review of these mechanisms is beyond the scope of this review, but multiplex T-cell engineering offers an opportunity to influence T-cell fate and to prevent or correct the development of T-cell dysfunction.

Once T cells or CAR-T cells recognize their antigen and receive proper activating signals, they undergo various transcriptional, epigenetic, and metabolic changes that commit them to different fates, from early memory (stem cell memory T cells - Tscm or central memory T cells - Tcm) to effector memory (Tem) to terminally differentiated effector T cells (Teff). Early memory T cells are long-lived and have the capacity to self-renew, whereas Teff cells gradually undergo functional decline and eventually apoptosis. Several pre-clinical and clinical lines of evidence support that early memory T cells outperform Teff in adoptive immunotherapy, including the CAR-T cell field (109–117). Studies on CAR T cells have revealed the importance of activation signals in T-cell differentiation. The choice of co-stimulatory domain (CD28 *vs*. 41BB, for example) impacts memory fate and CAR T-cell efficacy in certain contexts, and CAR design that avoids tonic signaling prevents the development of T-cell dysfunction (118). Beyond receptor design, an ingenious strategy to skew therapeutic CAR-T cell differentiation towards a memory phenotype through advanced genome editing is to strike at the epigenetic level, allowing chromatin accessibility of the genes that regulate memory formation and lead to the acquisition of an early memory phenotype (119). As multiple inhibitors of T memory formation have been identified in the chromatin machinery, their deletion using CRISPR-Cas9 technology has shown impressive results in several studies. For example, CRISPR-Cas9 inactivation of the H3K9 trimethyltransferase SUV39H1 in CAR-T cells promoted a self-renewing and stem-like phenotype that allowed for the long-term persistence of CAR-T cells and protection against tumor relapse (120). In addition, deletion of *de novo* DNA methyltransferase 3 alpha (DNMT3A) provided overall resistance to CAR-T cell exhaustion, which exhibited enhanced proliferation, *in vivo* persistence, and tumor control in prolonged tumor exposure (121). Other promising targets include the master transcription factors of Teff fate, such as NR4A receptors (122) and PR domain zinc finger protein 1 (PRDM1), which encodes BLIMP-1. Disruption of PRDM1 using CRISPR-Cas9 promoted the expansion of less-differentiated memory CAR-T cells *in vivo* and enhanced T-cell persistence in multiple tumor models (119). Using a model of tonic CAR signaling leading to T-cell exhaustion, retroviral overexpression of c-Jun, an AP-1 factor, enhanced CAR T-cell expansion and functionality, decreased terminal effector differentiation, and improved antitumor potency in five different *in vivo* tumor models (123). Repeatedly stimulated and proliferating T cells eventually acquire features of cellular senescence (activation of the DNA damage response and cell cycle arrest, increased β-galactosidase activity, and dysfunctional mitochondria) (107). While often described as irreversible, subsets of T cells displaying cellular senescence features can be revived by targeting senescence-associated pathways, such as p38 MAP kinase and p16^Ink4^ (124–126). Repeated antigen exposure and suboptimal activation signals can also lead to T-cell exhaustion, characterized by decreased effector functions such as cytokine secretion and cytotoxicity, limited proliferation rate and self-renewal capacity, and upregulation of inhibitory co-receptors (or immune checkpoints) such as PD-1, TIM-3, LAG-3, and TIGIT, among many others (107). Several ligands for immune checkpoints and other inhibitory molecules are expressed by tumor cells and other cells within the tumor microenvironment. Therefore, T-cell exhaustion is a cardinal feature of cancer immunology, and immune checkpoint-blocking antibodies have become the standard of care for a wide spectrum of malignancies. The success of immune checkpoint blockade hinges on T-cell populations at the early stages of the exhaustion process (127, 128), and the blockade of PD-1 can temporarily revive exhausted T cells but may be unable to restore a memory phenotype (129). However, clinical trials using antibody-mediated PD-1 blockade in combination with CAR-T cells did not improve outcomes relative to those reported with CAR T cells alone (130, 131). In contrast, CRISPR-Cas9 mediated multi-editing of T cells for CAR expression and PD-1 inactivation has shown encouraging results in both preclinical (132) and clinical studies (133). Gene editing of other targets, such as LAG3 (134) and CTLA4 (135), blocks the suppressive signals from the tumor microenvironment and enhances the effector functions of CAR-T cells (116). Advances in gene-editing technology allow for the targeting of multiple immune checkpoints and can yield superior reinvigoration relative to single blockade in human T cells (136, 137). In addition to the strategy described above, which leverages Cas13-based methods to target multiple immune checkpoints, one study showed the possibility of efficient multiplex genomic editing of CAR T cells via a single CRISPR protocol by incorporating multiple gRNAs into a CAR lentiviral vector to target PD-1 and CTLA4 simultaneously (138). The same concept was applied to target PD1, TIM3 and LAG3 in CAR-T cells using short hairpin RNA cluster to enhance tumor control (136, 139–141). In another study, CRISPR/Cas9 RNP electroporation was used to knock out PD-1, LAG-3, and TIM-3 in CD8^+^ T-cells. Edited T cells demonstrated improved expansion and persistence in a mouse model, delayed tumor growth, and enhanced survival without added toxicity (142). Alternative strategies include targeting intracellular checkpoints, such as cytokine-inducible SH2-Containing Protein (CISH), or upstream regulators of multiple immune-checkpoint expressions (143, 144).

#### 3.1.2 Alloreactivity and drug resistance

A cardinal feature of T cells is the recognition of MHC-associated alloantigens and self from non-self. Therefore, adoptively transferred allogeneic T cells that retain the potential to recognize histocompatibility antigens pose a safety risk. Conversely, therapeutic T cells that are susceptible to immune rejection compromise the efficacy of cellular products. Multiplex engineering offers several strategies for the development of allogeneic T-cell therapy and the coherent integration of T-cell products into complex treatment schemes. The development of allogeneic T cell therapeutics is appealing for several reasons: T cells harvested from healthy donors are less dysfunctional than autologous T cells obtained from cancer patients, the manufacturing of large batches of allogeneic T cells is less costly per dose than autologous therapies, and “off-the-shelf”, ready-to-use cell therapies could lead to faster access for patients (8). The transfer of partially histoincompatible T cells has both advantages and disadvantages in the context of adoptive immunotherapy. While targeting alloantigens is a long-proven strategy to treat several blood cancers in the context of AHCT, either through unmanipulated or genetically modified T cells (4), histocompatibility is a barrier limiting the development of allogeneic T-cell therapies (4). Recognition of alloantigens on host cells by adoptively transferred T cells may result in graft-versus-host disease (GVHD), and allogeneic therapeutic T cells may be rapidly rejected by immunocompetent host T cells. A conceptually simple approach to CAR T cell therapy is to ablate or reduce the expression of genes responsible for TCR-MHC recognition of alloantigens (138, 145, 146). A recent study has shown the potential of multiplex editing for the optimization of therapeutic T cell products by simultaneously knocking out four genes (TRAC or CD3E, Beta-2 microglobulin - B2M, Class II Major Histocompatibility Complex Transactivator – CIITA, and Poliovirus receptor) to eliminate the risk of GVHD, as well as rejection by both T lymphocytes and NK cells (147). Gene editing was performed using two methods: CRISPR/Cas9 nuclease and adenine base editor (ABE). ABE-edited CAR-T cells showed higher manufacturing yields, superior *in vitro* effector functions under continuous antigen stimulation, reduced activation of p53 and DNA damage response pathways at baseline, improved tumor control, and extended overall survival compared to their Cas9-edited counterparts. This further emphasizes that, beyond the choice of genes to edit, the methods used for gene editing may be equally important to the success of therapeutic T cells.

T-cell therapies are increasingly used for complex therapeutic regimens and medical conditions. This suggests that optimal T cell function and efficacy may be affected by the concurrently administered drugs that affect T cell physiology. One of the classical indications for adoptive T-cell immunotherapy is the restoration of immunity in the context of post-transplant immunosuppression. Inactivation of the glucocorticoid receptor or FKBP12 has been shown to confer T-cell resistance to corticosteroids and tacrolimus, respectively, and as such, may be integrated into multiple types of T-cell therapies for patients requiring broad immunosuppression (148–150). Similarly, the deletion of CD52 expression protects engineered T cells against the ablating effects of the monoclonal antibody alemtuzumab, which is commonly used for a variety of indications, including T-cell depletion in AHCT (151). Suppression of cell surface receptor expression is also relevant for CAR T-cell therapy targeting T-lineage malignancies. The pan-T-cell markers CD5 and CD7 can be effectively targeted using anti-CD5 or CD7 CAR. To avoid T-cell fratricide, CD5 or CD7 can be edited in anti-CD5 or anti-CD7 CAR T cells. The loss of CD7 or CD5 does not compromise normal T-cell physiology and, in the case of CD5, may even be beneficial. The type I transmembrane glycoprotein CD5 is a negative regulator of TCR signaling, and recent evidence has revealed that CD5 deletion improves CAR T-cell efficacy in pre-clinical models (152).

### 3.2 Engaging cancer with multifunction and multi-edited T cells

Modulating intrinsic T-cell physiology, conferring drug resistance, and mitigating alloreactivity are relevant to the design of T-cell immunotherapies. However, optimized cellular immunotherapies must also consider the biology of cancer cells and their environments. This section reviews how multi-functional and multiplex T-cell editing can address the crucial issues of cancer cell immune escape, trafficking into tumor beds, and resistance to hostile tumor environments. Most cellular engineering designs used thus far rely on a single engineering step consisting of the viral transduction of vectors containing multiple genes, conferring multiple functions. However, multimodal (viruses and nucleases) and non-viral methods are increasingly being used. Early phase clinical trials are being conducted to test several of these strategies, with clinical results increasingly available (see Section 4 below).

#### 3.2.1 Avoiding antigen escape

Antigen loss is a common mechanism of tumor-immune resistance. Targeting a single antigen, whether MHC-associated or not, can lead to immune-mediated selection of resistant cancer cell variants. Several strategies can be considered for multi-antigen targeting in adoptive T-cell immunotherapy. Co-infusion (simultaneous or sequential) of multiple single-specificity T cells (TCR transgenic or CAR) appears safe and promising in clinical trials (153–156).In parallel, refinements in genetic engineering can confer multi-antigen specificity. In the CAR field. Several designs exist in the CAR field, including the co-expression of two distinct CARs (dual CARs), and the engineering of a single construct with two different single-chain variable fragments (scFv) (tandem CAR)(reviewed in (157)). The manufacture of CAR T cells from antigen-specific T cells offers the possibility of simultaneously targeting MHC-associated and MHC-independent antigens at the same time. Recent preclinical studies on dual targeting of acute myeloid leukemia with a transgenic NPM1-antigen-specific TCR and a CD33 CAR revealed that double transgenic receptor expression led to better cytotoxicity and tumor control relative to single receptor-expressing T cells (158). Although promising, this may not be applicable to all antigenic receptor pairings. The expression of two receptors may lead to reduced activity of one of the receptors, potentially affecting T-cell function and the efficacy of antigen recognition compared to single-specificity T cells. (159, 160). Immune-mediated selection of resistant cancer variants is a well-described phenomenon in the CD19 CAR T cell field, where several mechanisms for CD19 loss have been characterized, including point mutations, defective splicing, lineage switching, and epitope masking (161). Consequently, several groups have developed dual- or triple-expression CAR approaches to enable the simultaneous targeting of several B cell lineage antigens (CD22, CD20, and CD79a) (162–164) or combine CD19 targeting with antigens that are not strictly recognized as lineage-specific, such as CD123 or CD70 (165, 166). Likewise, several teams have devised multi-antigen targeting to circumvent the issue of B-cell maturation antigen (BCMA) loss of expression in multiple myeloma. Currently approved BCMA-directed CAR T cell products have provided impressive results but are not considered curative (167). The use of tandem CARs targeting BCMA and Transmembrane activator and CAML interactor (TACI) or BCMA and G protein-coupled receptor class C group 5 member D (GPRC5D) with a dual CAR approach in pre-clinical models showed better efficacy and reduced antigen escape (168, 169).

#### 3.2.2 Migration into tumors

One of the main limitations of adoptive T-cell immunotherapy, especially in solid tumors, is the inefficient trafficking and poor infiltration of these cells at the tumor site, as shown in multiple preclinical studies (170–172). Clinical evidence also suggests limited T-cell accumulation in some solid tumors, although detailed patient-level data remain limited (173, 174). The first strategy to address this issue is to equip therapeutic T cells with chemokine receptors that attract T cells to the tumor bed. Initial candidates for overexpression have been the chemokine receptors CCR2 and CXCR2 (receptors for CCL2 and CXCL8/IL-8, respectively) for CAR and transgenic TCR T cells, as well as TILs, which revealed increased tumor infiltration in several models (175–181). Other chemokine receptor strategies that target different axes relevant to the tumor microenvironment have been developed. Co-expression of CCR4 improved the homing ability of anti-CD30 CAR-T cells to Hodgkin tumor sites by enhancing their migration toward CCR4 ligands CCL17 and CCL22, which are highly expressed in the tumor microenvironment (182). The modification of CAR-T cells targeting the lung adenocarcinoma antigen MUC1 for the chemokine receptor CCR6 enhanced migration toward tumor sites rich in CCL20 and CAR-T cell efficacy (183). Other examples include T cells engineered to express the chemokine receptor CXCR5 to improve migration in CXCL13 rich lung as well as head and neck cancer microenvironments, and CXCR6 expression that improved migration and function in hypoxic CXCL16-rich pancreatic tumor milieus (184, 185). Finally, fractalkine (CX3CL1) offers another promising route to enhance CX3CR1-driven migration. Fractalkine, unlike many other chemokines, exists in both membrane-bound and soluble forms, creating an effective gradient to attract CX3CR1+ T-cells. In melanoma and pancreatic cancer models, T cells transduced with CX3CR1 demonstrated improved T-cell trafficking to tumors and inhibition of tumor growth (186). The chemokine paradigm can also be exploited using armored T cells engineered to secrete chemokines to attract other immune cells. CCL19 can attract dendritic cells and T cells into tumor beds and has been investigated in CAR T cells for solid tumors (187). In addition to altered chemokine cues, certain tumors have a dense and fibrotic extracellular matrix (ECM) that acts as a physical barrier to therapeutic T-cells. To overcome this obstacle, researchers have engineered CAR T cells to express ECM-degrading enzymes, such as heparanase, which targets and cleaves heparan sulfate proteoglycans, a key structural component of the tumor stroma. In a preclinical study, heparanase-expressing CAR T cells demonstrated significantly improved tumor infiltration and antitumor activity in solid tumor models, with no observed increase in off-target effects (188). Extending this strategy, CAR-T cells engineered to express mature metalloproteinase-8 (mMMP-8) showed enhanced infiltration into tumor tissues and improved antitumor efficacy by degrading collagen fibers within the extracellular matrix, thereby facilitating deeper tumor penetration (189). Furthermore, CAR-T cells secreting relaxin-2 demonstrated increased efficacy and infiltration in stromal-rich solid tumors by remodeling the tumor microenvironment and reducing fibrosis, ultimately promoting better T-cell migration and antitumor responses (190).

#### 3.2.3 Resisting the tumor microenvironment

Tumor cells, stromal elements, defective angiogenesis, and infiltrating immunosuppressive immune cells all contribute to making tumor microenvironments inhospitable through metabolites and a lack of nutrients, cytokines, and cell-cell contacts. Strategies to counteract these deleterious effects can be grouped into three categories: 1) changing the microenvironment through the secretion of immune-stimulatory or homeostatic cytokines (T cells redirected for universal cytokine-mediated killing -TRUCK) or other biomolecules, such as immune checkpoint blockers, cytokine traps, or bi-specific engagers; 2) making therapeutic T cells insensitive to inhibitory signals; or 3) engineering T cells to transform inhibitory interactions into immune-stimulatory signals through switch receptors (191–193).

Gain-of-function cytokines or other biomolecule secretions are usually achieved through viral transduction and can provide homeostatic, chemotactic, or immunostimulatory signals (comprehensively reviewed in (191)). Among the best described immunostimulatory cytokines used in TRUCKs are IL-12 and IL-18, which have been shown to remodel the tumor microenvironment in several experimental systems by increasing the infiltration of inflammatory immune cells, such as M1 macrophages, NK cells, and T cells. While constitutive IL-18 secretion appears manageable in clinical trials, constitutive IL-12 is toxic (see Section 3.3), leading to refinement in vector design to restrain IL-12 secretion to activated T cells. However, even when driven by a nuclear factor of activated T cells (NFAT) promoter, IL-12 secretion by T cells was toxic (194). Pre-clinical data suggest that insertion of IL-12 at the PDC1 (PD-1) locus could likewise restrict IL-12 secretion to antigen-experienced T cells and lead to more modest secretion relative to NFAT-driven expression (195). Homeostatic cytokines, such as IL-7, IL-15, and IL-21, may promote T-cell persistence and result in better outcomes in certain preclinical models (196). To confer multiple functions simultaneously, cytokine production can be combined with other modifications. For example, EGFRvIII-targeted CAR-T cells have been engineered to co-express IL-15, IL-18, and CXCR2 using gammaretroviral delivery. This enhances CAR-T cell migration, survival, and antitumor activity in breast cancer models by reducing exhaustion and apoptosis without causing toxicity (197). Several clinical studies using cytokine armoring are ongoing and will provide important insights into the impact of cytokine secretion and, hopefully, generate hypotheses for the next wave of therapeutic T-cell armoring. Evolution to improve cytokine signaling and the specificity of the response includes built-in CAR designs to incorporate signaling modules and transgenic orthogonal receptors devised to signal following the administration of synthetic cytokines that are otherwise incapable of signaling through natural receptors (198–200). Loss-of-function strategies can also be leveraged to improve T-cell therapy in the tumor microenvironment. Certain metabolites, such as adenosine, are present at high concentrations in neoplastic environments and exert immunoregulatory effects. Compared to pharmacological blockade or shRNA-mediated knockdown, CRISPR-Cas9-mediated deletion of the adenosine A_2_a receptor in CAR T cells improved therapeutic efficacy in preclinical cancer models (201). TGF-β is a pleiotropic key immunoregulatory cytokine in tumors. Dominant-negative receptors and gene-editing approaches have improved T-cell function in pre-clinical models and are good candidates for incorporation into multiplex engineering strategies (202–205). Adenosine and TGF-β are among the many soluble inhibitors found in cancer microenvironments, and future studies should address whether multiplexing resistance to these mediators enables further gains. Another approach is to subvert inhibitory signals using switch receptors. For example, fusing the extracellular domain of the TGF-β receptor (TGFBRI) with the intracellular portion of the co-stimulator 4-1BB or IL-2/IL-15 receptor results in resistance to the effects of TGF-β and improved antitumor efficacy (193, 206). The PD-1/CD28 switch receptors are based on this concept. Several preclinical models and emerging clinical data support that CAR T cells co-engineered for the expression of a PD-1/CD28 switch receptor improve the therapeutic efficacy of T-cell therapies (207–211). This principle is being expanded to other immune checkpoint receptors, such as TIM-3 and TIGIT (212, 213). Tools to improve therapeutic T cells in tumor microenvironments are diverse and are increasingly available for devising multiplex-engineering strategies.

### 3.3 Preventing excessive toxicities

CAR T-cell therapy has changed the treatment of hematologic malignancies; however, its clinical application is often hindered by various toxicities. Among the most common and severe adverse effects are cytokine release syndrome (CRS), marked by systemic inflammation, fever, hypotension, and the potential for multiorgan failure due to excessive cytokine secretion by CAR T cells and other immune cells (214). Neurotoxicity, also known as immune effector cell-associated neurotoxicity syndrome (ICANS), presents with neurological symptoms such as confusion, seizures, and encephalopathy, likely caused by endothelial activation and blood–brain barrier disruption (215). On-target/off-tumor toxicity occurs when CAR T cells attack healthy tissues expressing the target antigen, leading to collateral damage to healthy tissues. B-cell aplasia is a specific on-target toxicity observed with CD19-directed CAR-T therapies, causing the depletion of normal B cells and increased infection risk (216). Tumor lysis syndrome (TLS) may follow rapid tumor cell destruction, causing metabolic imbalances and renal impairment (217). Additionally, macrophage activation syndrome (MAS) or hemophagocytic lymphohistiocytosis (HLH) represents a severe hyperinflammatory state linked to CAR T-cell therapy (218). In solid tumors, direct organ damage caused by the expression of the target antigen by epithelial or stromal cells may lead to significant toxicity (219). Although less frequent, off-target toxicity arises from unintended gene editing or cross-reactivity and may harm non-target tissues (220–222).

**Figure 3:**
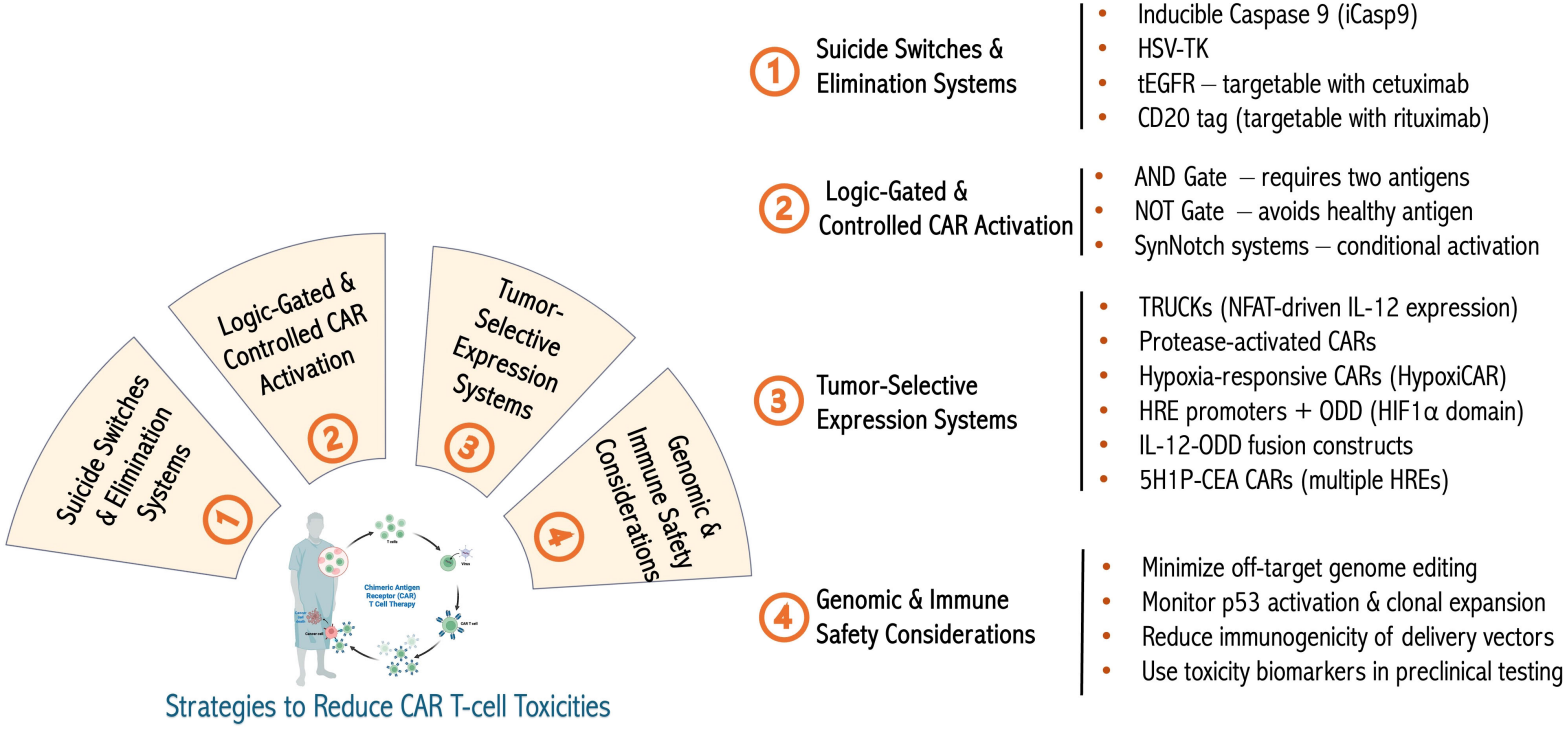
Advanced engineering to limit biological or genetic toxicities. Summary with examples of various approaches to limit the toxicity of genetically engineered therapeutic T cells as well as the genotoxicity and immunogenicity of cellular engineering methods. Figure constructed with visual elements from BioRender (https://BioRender.com/h5481mb).

Various safety strategies have been developed to reduce these risks (**Figure 3**). First, suicide switches, such as inducible caspase-9 (iCasp9), allow for rapid T-cell elimination upon administration of a small molecule and have been tested mainly in hematologic cancers (223). Clinical studies have validated iCasp9 in early phase trials, demonstrating reproducible elimination of infused cells and control of adverse events, with multiple dosing cycles feasible without cumulative toxicity (223, 224). Second, elimination markers, including truncated EGFR and CD20, provide targets for antibody-mediated depletion mechanisms (225, 226). Clinical evaluation of elimination markers has shown efficient CAR T cell depletion *in vivo*, confirming their potential to mitigate severe toxicities when necessary (227, 228). Next, logic-gated CARs that require dual antigen recognition enhance tumor specificity and reduce off-tumor effects (229). Preclinical validation of logic-gated CARs has demonstrated improved tumor selectivity and reduced off-tumor cytotoxicity, providing evidence of their translational potential (229, 230). Synthetic Notch (SynNotch) receptors enable the spatial restriction of CAR expression (231), whereas inhibitory CARs (iCARs) attenuate activation upon engagement with antigens on healthy tissues (232). Preclinical studies of SynNotch and iCAR systems have shown reduced systemic toxicity while maintaining antitumor efficacy (231, 232). Moreover, tunable systems, including tetracycline-responsive promoters and small-molecule “ON-switch” CARs, offer external control of CAR activity (233, 234). ON-switch CARs have been functionally validated in preclinical models, showing controlled T cell activation and mitigation of cytokine-mediated toxicity (235, 236). Additionally, tumor-selective protease-activated CARs and activation-inducible TRUCKs (for IL-12 production for instance as described above) confine potentially toxic cytokine secretion to the tumor microenvironments, and similarly, promoters such as NR4A2 and RGS16 have been designed to drive CAR or cytokine expression specifically in the tumor microenvironment, ensuring that the transgene is predominantly active only in tumor tissue, thereby enhancing safety and minimizing off-tumor effects (194, 237–240). Clinical or preclinical validation of TRUCKs and promoter-restricted CARs has demonstrated reduced off-tumor activity and lower systemic cytokine release (240–243). Finally, dual CAR systems, multi-step activation designs, and so-called masked CARs that rely on intra-tumoral proteolytic removal of a peptide blocking the CAR’s antigen-binding site for localized activation further restrict activation to malignant contexts (229, 239).

The tumor environment can be further leveraged through metabolic switches. The best example of this is hypoxia (244). For example, the incorporation of hypoxia-responsive elements (HREs) within transgene promoters, combined with oxygen-dependent degradation domains (ODDs) derived from HIF1α, enables selective transgene expression and stabilization only under low-oxygen conditions, which are prevalent in tumors. Hypoxia-sensing CAR T cells (HypoxiCAR) have demonstrated significant mitigation of systemic toxicities while preserving robust antitumor efficacy in preclinical models (244, 245). While clinical data are still emerging, preclinical studies have confirmed that HypoxiCAR significantly reduces systemic cytokine release and off-tumor toxicity while retaining antitumor activity (246, 247). Similarly, CAR-T cells engineered with multiple HREs in their promoters (e.g., 5H1P-CEA CARs) showed enhanced tumor specificity and reduced activity in normoxic environments, leading to improved safety profiles (248). Furthermore, the fusion of cytokines, such as IL-12, to ODD domains (CAR19/hIL12ODD) ensure controlled cytokine release restricted to hypoxic tumor microenvironments, minimizing systemic inflammatory side effects (249). These innovations complement existing molecular safety switches and underscore the potential of microenvironment-responsive CAR designs to optimize therapeutic windows and reduce adverse events.

Preclinical development should incorporate sensitive biomarkers to detect early signs of toxicity and evaluate next-generation platforms in the context of tumor heterogeneity, antigen escape, and the tumor microenvironment. Collectively, these strategies build a safety framework that is necessary to optimize both the efficacy and tolerability of engineered CAR T-cell therapies.

### 3.4 Integrating T-cell biology and tumor biology into therapies

Several T cell therapies have integrated many of the concepts described above into single T cell products. Representative examples that led to the clinical trials summarized in the next section illustrate the vast potential of contemporary cellular engineering methods.

Using a combination of viral and non-viral gene engineering approaches, several groups have pursued the objective of creating safe, rejection and fratricide-resistant, allogeneic “off-the-shelf” CAR T cells to treat T-cell leukemia. Targeting the universal T-cell marker CD7, this strategy involves the expression of anti-CD7 CAR through lentiviral transduction, CRISPR-Cas9 for CD7, and TCR alpha chain (TRAC) ablation to avoid fratricide and GVHD, respectively (250). This approach has been refined with the use of base editing and the addition of CD52 deletion to evade alemtuzumab-based lymphodepletion and has been tested in early phase clinical trials (251, 252). Base editing technologies have high potential for multiplex editing of therapeutic T cells. It was recently demonstrated that the simultaneous knock-in of a CAR transgene and knockout of four genes, B2M, to suppress MHC class I expression, CD52, TRAC, and PD-1, could be achieved without genotoxicity (253). Pushing even further, it is reported that a combination of adenine base editing and Cas12b nuclease could generate “stealth” knock-in CAR T cells resistant to allorejection, GVHD, adenosine, PD-L1, and TGF-β through the editing of B2M, CIITA (to suppress MHC-class II expression), CD3E (to suppress TCR expression), Adenosine A2a receptor (ADORA2A), PD-1, and TGFBR2 genes (254). Along the same lines, other groups used either non-viral or viral (transduction of multiple sgRNAs) to perform multi-editing in CAR T cells, similarly focusing on the ablation of the TCR/MHC axis and immune checkpoints (138, 255, 256). Multiplex editing has also been applied in transgenic TCR therapy. Deletion of TIM-3, LAG-3, and 2B4 genes by CRISPR-Cas9 led to superior functionality and resistance to exhaustion of transgenic TCR NY-ESO-1-specific T cells in preclinical myeloma models. The modular nature of electroporation-delivered CRISPR-Cas9-sgRNA complexes allowed for the comparison of single versus multiple edits, outlining the role of each immune checkpoint molecule (257). Taken together, multiple studies support multiplex editing as a reliable and clinically applicable approach for adoptive T-cell immunotherapy.

## 4 Multi-engineered T cells in the clinic

Despite the rapidly growing corpus of preclinical data on multiplex-engineered or multifunctional T cells, and several ongoing clinical trials, a relatively limited number of clinical study results have been published. This is especially true for clinical studies investigating engineered T cells using more than one method or at different gene loci (summarized in **Table 2**).

**Table 2:** Summary of clinical studies involving multiplex-edited T cells.

Disease	Engineering approach	Number of patients baseline disease status	Clinical and safety outcomes	Ref
Myeloma, Lipo-sarcoma	Autologous T cells Transgenic TCR-lentivirus CRISPR-Cas9 editing of: TRAC/TRBC, PD-1	3 adults Advanced disease, multi-refractory	Best response: stable disease in 2/3 patients, with secondary progression in one (liposarcoma). Persistence of T cells: 3–9 months Grade 3–4 cytopenia, no other high-grade adverse events Chromosomal translocation detected, no functional consequence	(260)
B-ALL	Allogeneic T cells Dual CD19/CD22 CAR-lentivirus CRISPR-Cas9 editing of: TRAC, CD52	6 adults Advanced disease, 2–8 previous lines of therapy, 4/6 with bone marrow blast count ≥50% at enrolment.	5/6 complete response at day 28, 3/5 responders MRD-negative at 4.3 months (longest follow-up: 228 days). Median persistence of T cells: 42 days All patients had CRS (grade 1-3), 3/6 patients had grade 3 infections No genotoxicity	(288)
Renal cell carcinoma	Allogeneic T cells CD70 CAR insertion in TRAC locus – CRISPR-Cas9 and AAV vector CRISPR-Cas9 editing of: CD70, B2M. (CTX130) cells	16 adults Metastatic disease, 1–6 previous lines of therapy.	ORR 33%, PFS 2.9 months. One durable complete response (>36 months). CTX130 persistence: 28 days CRS (grade 2 maximum) at highest cell doses. 3/16 had grade 3 infections	(289)
T-ALL	Allogeneic T cells CD7 CAR - lentivirus Base editing of: TRBC, CD52, and CD7	3 pediatric patients Active disease at time of treatment, multiple lines of prior therapies	Response evaluated at day 28 – 2/3 achieved MRD-negativity and proceeded to AHCT. Persistence of T cells at day 28 and until 1 month post AHCT. High grade cytopenia and CRS 1/3 fatality due to infection	(252)
NHL	Autologous T cells CRISPR-Cas9, one-step CD19 CAR (linear double strand DNA) insertion at the PD-1 gene locus	21adults Relapsed/refractory disease >90% stage III-IV	ORR 100%, PFS 19.5 months. 18/21 complete remissions, 11 relapses (3–21 months post treatment) Maximal CRS – grade 2, limited ICANS Persistence until day 125 shown for 3 patients 19-30% CAR integration, 80-90% PD-1 locus edited. Off target locus identified, no functional consequences	(132, 290)
B-ALL	Allogeneic T cells CD19 CAR – lentivirus CRISPR-Cas9 editing of: TRAC, CD57	6 pediatric patients Relapsed/active disease at treatment	4/6 patients reached complete remission and bridged to AHCT (2 relapsed after AHCT), 2/6 had progressive disease correlated with poor therapeutic T-cell expansion Persistence at day 28 for 2/6 No high-grade CRS, one case of severe neurotoxicity, high-grade cytopenia in all patients. Translocation and off-target genetic editing estimated at < 1%	(291)
Peripheral and cutaneous T-cell lymphoma	Allogeneic T cells CTX130 T cells (as above)	39 adults Relapsed/refractory disease Average of 4 previous therapies	ORR 46%, 32% complete responses (including one persisting at 12 months with no other treatment) No CTX130 persistence beyond day 28 for most patients Grade 3–5 side effects included neutropenia (36%), infections (26%), cardiac failure (6%), hemophagocytic lymphohistiocytosis (8%) and CRS (3%)	(292)
B-ALL	Allogeneic T cells CD19 CAR -lentivirus TALEN editing of: CD52, TRAC	21 (7 pediatric, 14 adult patients) Median of 4 previous lines of treatment (62% had previous AHCT)	ORR 67%, 71% of responders MRD negative, 71% of patients bridged to AHCT Median duration of response 4.1 months, PFS at 6 months 27%. Persistence of T cells less than 28 days except for three patients (2 >42 days, 1>120 days) Grade 3–5 side effects included CRS (14%) infections (39%, including two deaths), cytopenia (75%, day 28 – 52% day 42).	(293)
Mesothelin-expressing neoplasms	Allogeneic T cells Mesothelin CAR - lentivirus CRISPR-Cas9 editing of: TRAC, PD-1	15 adults 8 different cancer histologies Median of 10 previous therapies	Best response – stable disease in 7/15 (3–4 weeks) and 2/15 (8–12 weeks). PFS for stable disease, 7.1 weeks. CAR T-cell persistence: 1 month Low rates of CRS, possible on-target toxicity (pleural, pericardial effusions, ascites) in 3/15 patients.	(133)
B-ALL, NHL	Allogeneic T cells CD19 CAR -lentivirus CRISPR-Cas9 editing of: TRAC, B2M, HLA-A/B	9 patients (16–65 years old) Active relapsed refractory disease	First 6 patients –B2M editing: no measurable therapeutic effect following B2M ablation (evidence of NK cell rejection). Limited expansion/persistence 3 patients –HLA-A/B editing: improved expansion, suggestion of improved anti-neoplastic activity Grade 3–5 cytopenia, no other significant side effects	(258)
NHL	Allogeneic T cells CD19 CAR + CD20 safety switch - lentivirus TALEN mediated editing of: CD52, TRAC deletion	33 adults Relapsed/refractory Median of 3 previous lines of therapy	ORR 19/33 including 14 complete response (8/33 at 12 months). Duration of response: 11.1 months Improved efficacy with higher doses. CAR T-cell persistence up to 4 months Grade 3–5 side effects in 94% including cytopenia (up to 82%), CMV reactivation (12%).	(294)

B or T-cell lymphobastic leukemia (B-ALL, T-ALL), non-hodgkin lymphoma (NHL). T-cell receptor (TCR). Chimeric Antigen Receptor (CAR). Clustered Regularly Interspaced Short Palindromic Repeats (CRISPR). T Cell Receptor Alpha Constant (TRAC), T Cell Receptor Beta Constant (TRBC). Beta-2 microglobulin (B2M). Programmed death-1 (PD-1). Adeno-associated virus (AAV). Transcription Activator-Like Effector Nuclease (TALEN). Human Leucocyte Antigen (HLA). Minimal residual disease (MRD), cytokine release syndrome (CRS), overall response rate (ORR), progression-free survival (PFS).

### 4.1 Overview of clinical trials using multi-edited T cells

To date, the published clinical data on multiplex-edited therapeutic T cells principally relate to two key concepts we previously described: resistance to exhaustion through the editing of immune checkpoint genes and the avoidance of alloreactivity, especially to facilitate the persistence of allogeneic products.

Although all early phase, highly heterogeneous (engineering approaches, diseases, allogeneic *vs*. autologous, number and impact of previous or concomitant therapies, multiple dose levels, etc.) and generally including small numbers of patients, these clinical studies offer crucial insights. Many pioneering studies have included detailed assessments of genotoxicity, which are overall reassuring for both CRISPR-Cas9 and base editing approaches. Although off-target editing and chromosomal anomalies have been reported in some studies, no functional impact has been noted. While firm conclusions about safety will require a longer follow-up (few studies report outcomes beyond a few months), these early results support the further development of advanced engineering methods. Compared to standard CAR T cells, multiplex-edited CD19 CAR T cells appear to confer a similar risk of adverse events, such as CRS and cytopenia, the latter being largely attributed to the lymphodepleting regimen. Similarly, early evidence of efficacy is difficult to interpret in the absence of a control group, which is expected in phase I-II or proof-of-concept studies. As previously reported, hematological cancers respond better to T-cell therapy than solid cancers. Other currently investigated designs, including TRUCK or migration-enhanced T cells (as described above), may improve the response in solid cancers and feed a new wave of multiplex engineered T cells in solid tumors (as discussed in Section 4.2). A note of caution regarding allogeneic products is the relatively limited persistence of engineered cells, as reported in several studies. Suppression of all MHC I molecule expression through B2-microglobulin editing may be conducive to NK cell-mediated rejection to a greater extent than selective HLA-A/B editing (as (258)). Preclinical studies suggest that other strategies to mitigate allogeneic T cell rejection by NK cells include the ablation of the adhesion ligands CD54 and CD58 and may be considered in multiplex engineering designs (195). Multi-engineered T cells may have a survival disadvantage in certain settings. Loss of TCR expression has been shown to affect persistence in one of the studies (133). Successful TRAC-edited mesothelin CAR T cells did not persist as long as unedited T cells, suggesting a plausible homeostatic role for TCR signaling (259). In addition, PD-1 editing may precipitate T-cell dysfunction and loss, as suggested in the first study reporting on multiplex and CRISPR-Cas9 engineered T cells in human (260), and in line with a previous study in PD-1 knock-out mice (261). Hence, multiplex engineering allows for the counteraction of certain constraints of T-cell immunology but may unveil both predictable and unsuspected vulnerabilities.

### 4.2 Multiplex editing to address the challenge of solid tumor T-cell immunotherapy

Long-term remissions, complete responses, or bridging to potential curative therapies following engineered T-cell administration, have mostly been described for hematopoietic neoplasms. This is also true for multiplex-edited T cells and approaches targeting multiple antigens. Although not thoroughly discussed here because the therapeutic T cells tested had not undergone multiplex editing, recently published clinical studies investigating CRISPR-Cas9 edited cancer patient T cells or TILs from cancer patients are reassuring about the feasibility and safety of gene-engineered T cells. In the first study, lung cancer patients received autologous peripheral blood PD-1 edited T cells manufactured from peripheral blood (262). Detailed analysis revealed no major genotoxicity, and the treatment was well tolerated. In a landmark study that included clinical results, autologous NY-ESO-1 transgenic TCR T cells were generated following lentivirus delivery to patient T cells previously edited at the TRAC and TCR beta chain (TRBC) gene loci to avoid TCR chain mispairing and at the PDCD1 (PD-1) locus using the CRISPR-Cas9 approach (260). In another study using gammaretrovirus-mediated neo-antigen-specific TCR transgenic expression in patients with metastatic colon cancer, three out of seven patients had objective responses (156). Multiantigen targeting with personalized neoantigen-specific TCR is thus feasible but poses significant financial and logistical challenges that may be partly alleviated with non-viral methods. Such strategy was used in another trial, where personalized neo-antigen-specific TCR were inserted *in situ* at the TRAC locus and reinjected in patients with metastatic cancer. Approximately one-third of patients had stable disease following treatment, and no significant toxicity was observed (155). The discovery of “public” cancer-specific antigens, such as KRAS^G12V^ will further facilitate the design of multi-antigen targeting in solid tumors (263–265). In lymphoblastic leukemia, patients who received CD19/CD22 (tandem or sequential) had a higher complete response and minimal residual disease-negative rates than those who received CD19 CAR T cells alone (266). Although additional evidence will be required to firmly conclude on the efficacy of the various CD19/CD22 approaches (267, 268), the concept of multi-antigen targeting using CAR T cells may be translated to solid tumors with recent pre-clinical data suggesting that a tandem mesothelin-MUC16 CAR is superior to monospecific CARs (269).

Other improvements in TILs have also been tested in the clinic. The production and injection of CISH-deleted gastrointestinal tumor-derived TILs has recently been reported. Gene-edited TILs were successfully manufactured in 86% (19/22) of the recruited patients, and 12 patients were treated. Side effects were as expected and unrelated to the TIL product; six patients had stable disease, and one patient with microsatellite instability achieved a complete response (144). Other approaches are currently under clinical investigation, including PD-1 deletion and cytokine signaling modulation (dominant-negative TGF-β receptor, etc.) to improve T-cell function, as well as several armored T-cell products aimed at altering the hostile tumor microenvironment (as described above), but very limited published clinical data are available.

Although disappointing compared to the results in hematopoietic neoplasms, engineered T-cell therapies for solid tumors are feasible. It is to be expected that clinical trial designs will have to account for the multi-layered complexity of solid tumors by enabling the simultaneous and coherent targeting of multiple antigens, intrinsic T-cell dysfunction and extrinsic constraints of the tumor microenvironment. While multiplex T-cell engineering can address some of this complexity, multi-product treatment schemes and optimization of the integration of cellular products relative to other treatments (timing, repeated dosing, etc.) will be required.

## 5 Conclusions and perspectives

This review focuses on the concepts and methodologies underlying the development of multifunctional and multiplex-edited T cells in the context of adoptive immunotherapy for cancer and emphasizes the rationale behind pioneering early clinical studies in the field. Other developments, such as *in vivo* gene editing, will also expand the field of cancer immunotherapy but would require a dedicated review. The development of therapeutic T-cell products, enhanced through multi-engineering and/or capable of meditating several different functions, is progressing rapidly. Although it was impossible to describe all the work being done in the field, the master principles driving the evolution of T-cell adoptive immunotherapy have remained centered on a few key concepts: These include the preservation of T-cell fitness, attempts to override tumor escape mechanisms, avoidance of toxicities, and adaptations required to perform in certain clinical situations (allogeneic therapy, concomitant drug use, etc.). However, there has been a noticeable shift from polycistronic viral vectors to more modular approaches using several different methodologies (viral and non-viral) to perform multiple gene editing. Contemporary gene-editing methods offer flexibility and new capabilities for performing multiplex gene modifications. Whether the future will see the replacement of virus-mediated genetic engineering by non-viral gene-editing methods or co-existence is unclear at this stage. Nevertheless, ever-improving technologies for genetically engineering immune cells have paved the way for the rapid clinical development of enhanced T-cell therapeutics. As for other cell therapies, this development will have to be matched with increased capacity in GMP-reagent manufacturing and cell production, as well as a rapidly adapting regulatory environment to enable the conduct of clinical studies and eventual incorporation into the standard of care (270).

To that effect, this review focused on the biological rationales for multi-editing and the remarkable innovations that made this possible, but we recognize that an equal challenge will be to implement these elaborate and costly therapies. Therefore, innovations in process development and implementation are required to sustainably translate advanced cell therapies. Contemporary manufacturing reviews consistently identify lentiviral vector (LVV) production and release testing as persistent bottlenecks due to multi-plasmid upstream complexity, stringent analytics, and constrained global capacity, even as newer producer-cell-line platforms improve yields (271–273). Two complementary strategies have gained traction to mitigate these pressures. First, closed, automated manufacturing improving reproducibility and possibility to manufacture in both centralized and point-of-care settings (272, 274–276). Second, non-viral or reduced-viral gene-transfer/editing approaches (transposon systems such as Sleeping Beauty or piggyBac; CRISPR RNP electroporation) remove or downsize the reliance on LVV. These platforms can compress manufacturing timelines and alleviate vector-related cost pressures while maintaining product potency (277, 278). In addition to manufacturing, quality control analyses and quality assurance systems remain complex and costly, requiring innovations to facilitate clinical translation/implementation without compromising safety. Together, non-viral editing plus closed-system automation constitutes a pragmatic path to industrialization one that improves scalability and reproducibility while directly addressing the economic bottlenecks documented across centers (271, 279–281).

The main objective of this review was to highlight how genetic engineering of T cells translates our notions of T cell and tumor biology into therapies. However, cellular engineering of other cell types has the potential to significantly improve adoptive cancer immunotherapy. Therapeutic NK cells benefit significantly from multi-engineering, particularly through the expression of homeostatic cytokines for expansion and persistence (282), enabling a vast arsenal of NK-based therapies (reviewed in (282–284)). Among these, NK-cell and other multiplexed engineered immune cells can be derived from induced pluripotent stem cells (285). Another layer of innovation involves the combination of engineered T cells with other cell types. For example, data from several groups suggest that the editing of CD33 in stem cells enables the use of anti-CD33 CAR T cells to treat acute myeloid leukemia in the context of AHCT. In this case, both normal myeloid precursors and leukemia cells express CD33, limiting the use of CD33-directed therapies. Shielding the transplanted stem and progenitor normal myeloid cells through the ablation of CD33 expression enables the normal restoration of myelopoiesis despite the co-administration of anti-CD33 CAR T cells to treat residual leukemia cells (286, 287). Hence, advanced therapeutic products could evolve toward the inclusion of multiple cell types engineered differently and co-administered. At present, it is unclear whether the simultaneous or sequential co-infusion of single-specificity T cells (e.g. CD19 and CD22 CAR T cells) will be superior or inferior to dual-specificity cellular therapeutics. Hence, whether multi-engineering of single cells will provide “all in one” packages to treat more effectively defined cancer types than combination therapies including one or several immune cell types carrying single modifications remains to be proven in clinical trials. Although the combination of several distinct immune cell products allows for flexibility in treatment schemes, multi-edited T cells may have an advantage in terms of regulatory compliance (i.e., validation of a single product versus several). The number of concepts and potential cell products to be tested is increasing and will require carefully designed clinical trials.
